# Supplementary material for: Histone H1 facilitates restoration of H3K27me3 during DNA replication by chromatin compaction
Source: Nat Commun. 2023 Jul 10;14:4081. doi: 10.1038/s41467-023-39846-y (PMC10333366; doi:10.1038/s41467-023-39846-y)
Supplement: Supplementary file 1 — Supplementary Information [file 41467_2023_39846_MOESM1_ESM.pdf]

Supplementary Information

Histone H1 facilitates restoration of H3K27me3 during DNA replication by chromatin compaction

Cuifang Liu[1,#], Juan Yu[1,#], Aoqun Song[1,2,#], Min Wang[1], Jiansen Hu[3], Ping Chen[1,4], Jicheng Zhao[1,*] and Guohong Li[1,2,5,*]

[1]National Laboratory of Biomacromolecules, CAS Center for Excellence in Biomacromolecules, Institute of Biophysics, Chinese Academy of Sciences, Beijing 100101, China;

[2]University of Chinese Academy of Sciences, Beijing 100049, China;

[3]Laboratory of RNA Biology, Institute of Biophysics, Chinese Academy of Science, Beijing 100101, China;

[4]Department of Immunology, School of Basic Medical Sciences, Beijing Key Laboratory for Tumor Invasion and Metastasis, Capital Medical University, Beijing 100069, China;

[5]Hubei Key Laboratory of Cell Homeostasis, College of Life Sciences, TaiKang Center for Life and Medical Sciences, Wuhan University, Wuhan 430072, China.

[#]These authors contributed equally to this work.

[*]Correspondence: zjch@ibp.ac.cn (J.Z.) and liguohong@ibp.ac.cn (G.L.)

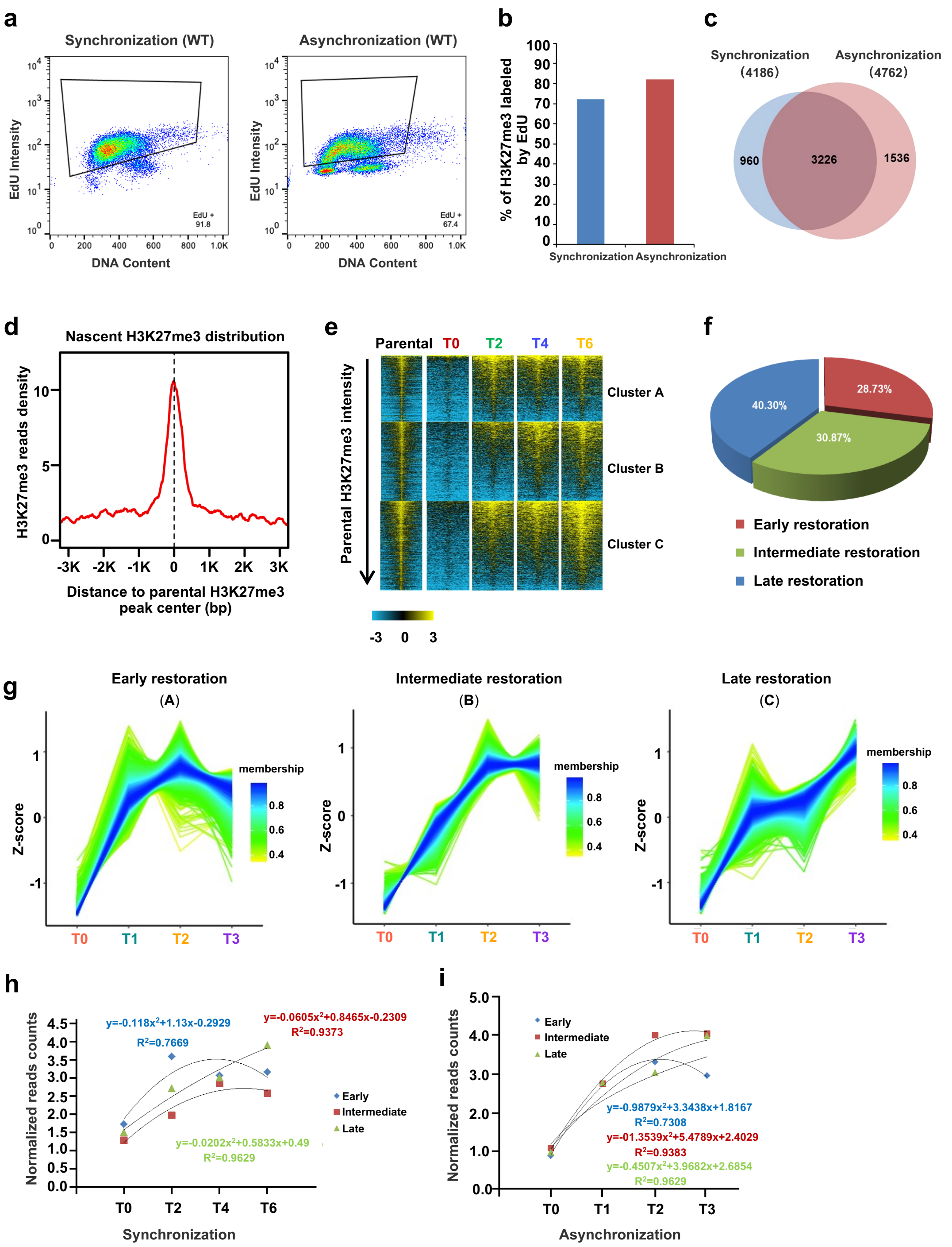

Supplementary Fig.1 H3K27me3 exhibits faster restoration at the dense repressive regions post DNA replication.

(**a**) Representative flow cytometry profiles of EdU-labeled cells in nascent samples of synchronized and asynchronous mESCs. The ChOR-Seq assays in asynchronous mESCs were performed twice. (**b**) Bar plots showing % of H3K27me3 peaks labeled by EdU versus total H3K27me3 peaks in synchronized and asynchronous mESCs in

the ChOR-seq experiments. (**c**)Venn diagram showing the overlapping of EdU-labeled H3K27me3 peaks in synchronized (blue) and asynchronous (orange) mESCs. (**d**) ChIP-seq cumulative enrichment of nascent H3K27me3 deposition, centered at the peak summit of parental H3K27me3 (±3 kb). Signal is quantitated using reference-adjusted reads per kilobase per million (RPKM). (**e**)Heat map analysis of H3K27me3 ChOR-Seq signals across H3K27me3-enriched regions (±3 kb) at different time points post-replication among clusters A-C, ranked from the highest to the lowest ChIP-Seq signals in mESCs. Colour intensity represents normalized and globally scaled tag counts. (**f**) Pie chart showing the percentage of the H3K27me3 clusters (early, intermediate and late restoration) in the EdU-labeled H3K27me3 peak regions. (**g**) Clusters of the restoration pattern of H3K27me3 in the asynchronous mESCs at the EdU-labeled H3K27me3-enrichment peak regions using time series cluster analysis. (**h-i**) Mathematical model for the restoration of H3K27me3 in synchronized (h) and asynchronous (i) mESCs among clusters A-C. Signal is quantitated using reference-adjusted reads per kilobase per million (RPKM). Source data are provided as a Source Data file.

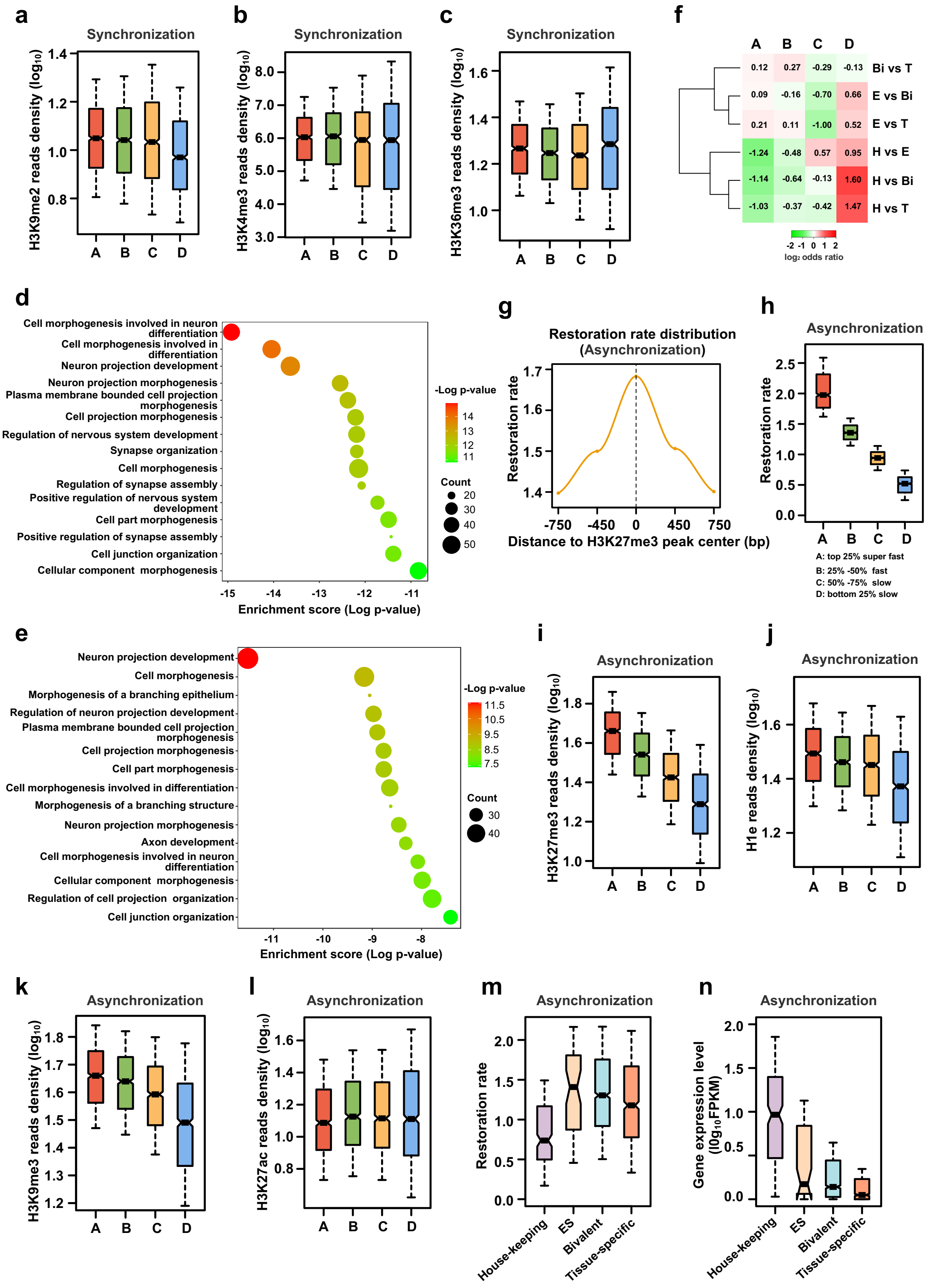

Supplementary Fig. 2 The restoration rate of H3K27me3 is positively corelated with repressive chromatin states.

(**a-c**) Boxplots showing the average reads density of H3K9me2 (a), H3K4me3 (b) and H3K36me3 (b) among cluster A-D in the synchronized mESCs (*n*= 1046 for A, *n*= 1047

for B, *n*= 1046 for C, *n*= 1047 for D). Signal is quantitated using reads per kilobase per million (RPKM). (**d, e**) GO enrichment analysis of the genes in cluster C (d) and cluster D (e). (**f**) Heat map showing the significance of the representation among different gene functional groups (relative to Fig.2j). Colour intensity represents $\log_2$ odds ratio. (**g**) Average profiles of restoration rate of H3K27me3 in the asynchronous mESCs across ± 750 bp EdU-labeled H3K27me3 peak center. Calculated using 1.5 kb windows with a 300 bp step, please see details in Methods section. (**h**)Boxplot showing the clusters of EdU-labeled H3K27me3 peaks in the asynchronous mESCs according to its restoration rate (*n*= 1115 for A, *n*= 1116 for B, *n*= 1115 for C, *n*= 1116 for D). (**i-l**) Boxplots showing the average reads density of H3K27me3 (i), H1e (j), H3K9me3 (k) and H3K27ac (l) among cluster A-D in the asynchronous mESCs (*n*= 1115 for A, *n*= 1116 for B, *n*= 1115 for C, *n*= 1116 for D). Signal is quantitated using reads per kilobase per million (RPKM). (**m, n**) Boxplot showing the restoration rate of H3K27me3 (m) and the gene expression levels (n) among different gene functional groups in the asynchronous mESCs (*n*= 200 for House-keeping genes, *n*= 49 for ES genes, *n*= 1488 for Bivalent genes, *n*= 881 for Tissue-specific genes). FPKM: fragments per kilobase of transcript per million fragments sequenced. The box plots (a-c, h-n) include the median line (median value indicated), the box denotes the interquartile range (IQR), whiskers denote the rest of the data distribution, and outliers are denoted by points greater than $\pm 1.5\times$IQR. Source data are provided as a Source Data file.

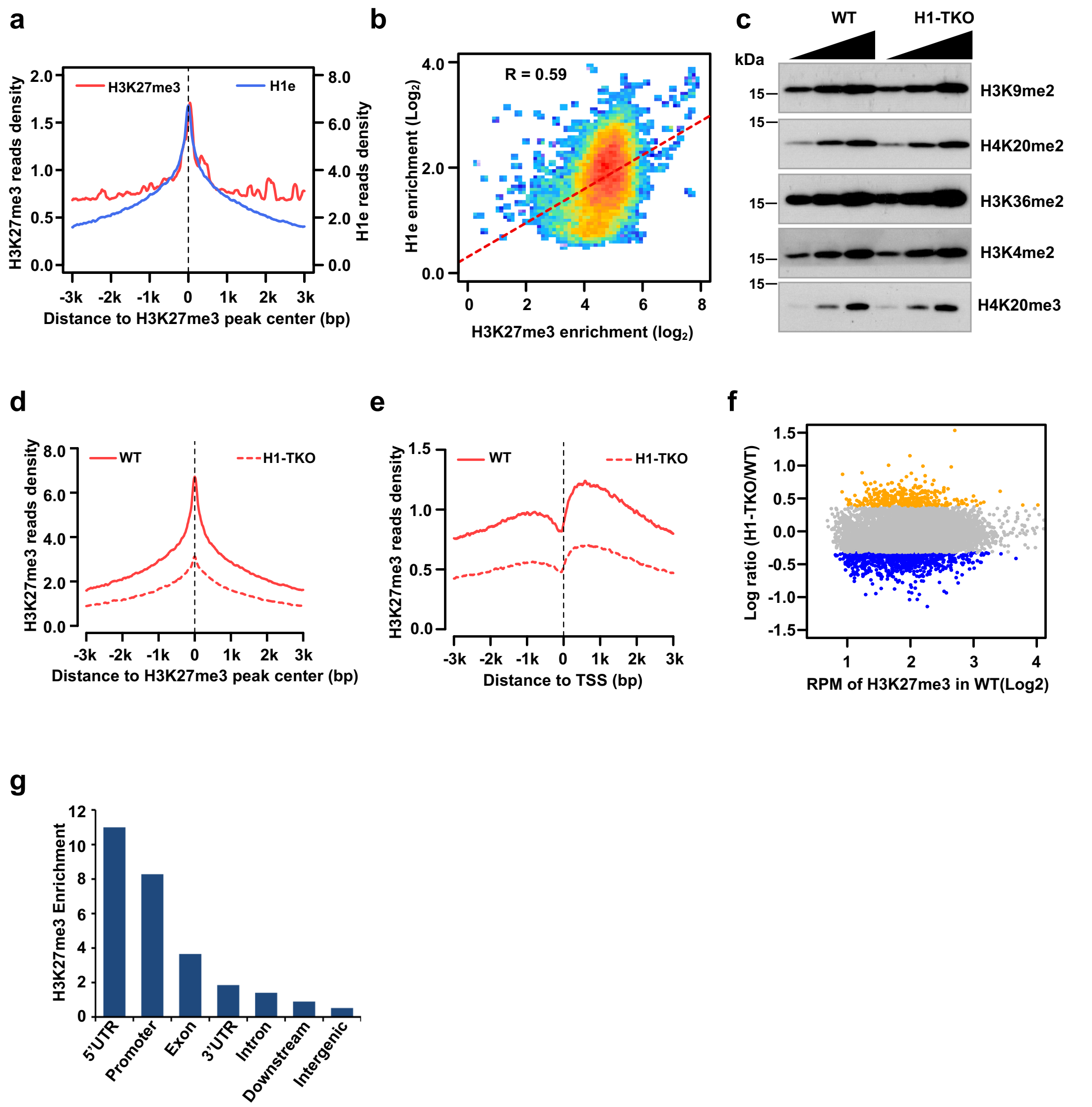

Supplementary Fig.3 The linker histone H1 plays an important role in maintaining H3K27me3 levels in mESCs.

(**a**) Average profiles of H3K27me3 and H1e ChIP-seq signal densities centered at the peak summit of H3K27me3 (±3 kb). Signal is quantitated using reference-adjusted reads per kilobase per million (RPKM). (**b**)Scatter plot of the Pearson's correlation between the H3K27me3 and H1e peak read densities in mESCs. The results are representative of two independent biological samples. (**c**)Representative immunoblots of various histone modifications in wild type and H1-TKO mESCs. The results are representative of *n*= 3 biologically independent experiments. Triangles represent concentration of proteins from low to high. (**d**)Average profile of H3K27me3 ChIP-seq signal densities centered at the peak summit of H3K27me3 in wild type and H1-TKO

mESCs (±3 kb). (**e**) Average profile of H3K27me3 ChIP-seq signal densities centered at the TSS regions (±3 kb) in wild type and H1-TKO mESCs. (d) and (e) are quantitated using reference-adjusted reads per kilobase per million (RPKM). (**f**) A scatter plot showing the change of H3K27me3 reads density (H1-TKO vs wild type mESCs). (**g**) Genomic distribution of the reduced H3K27me3 signals after partial H1 knock out. Source data are provided as a Source Data file.

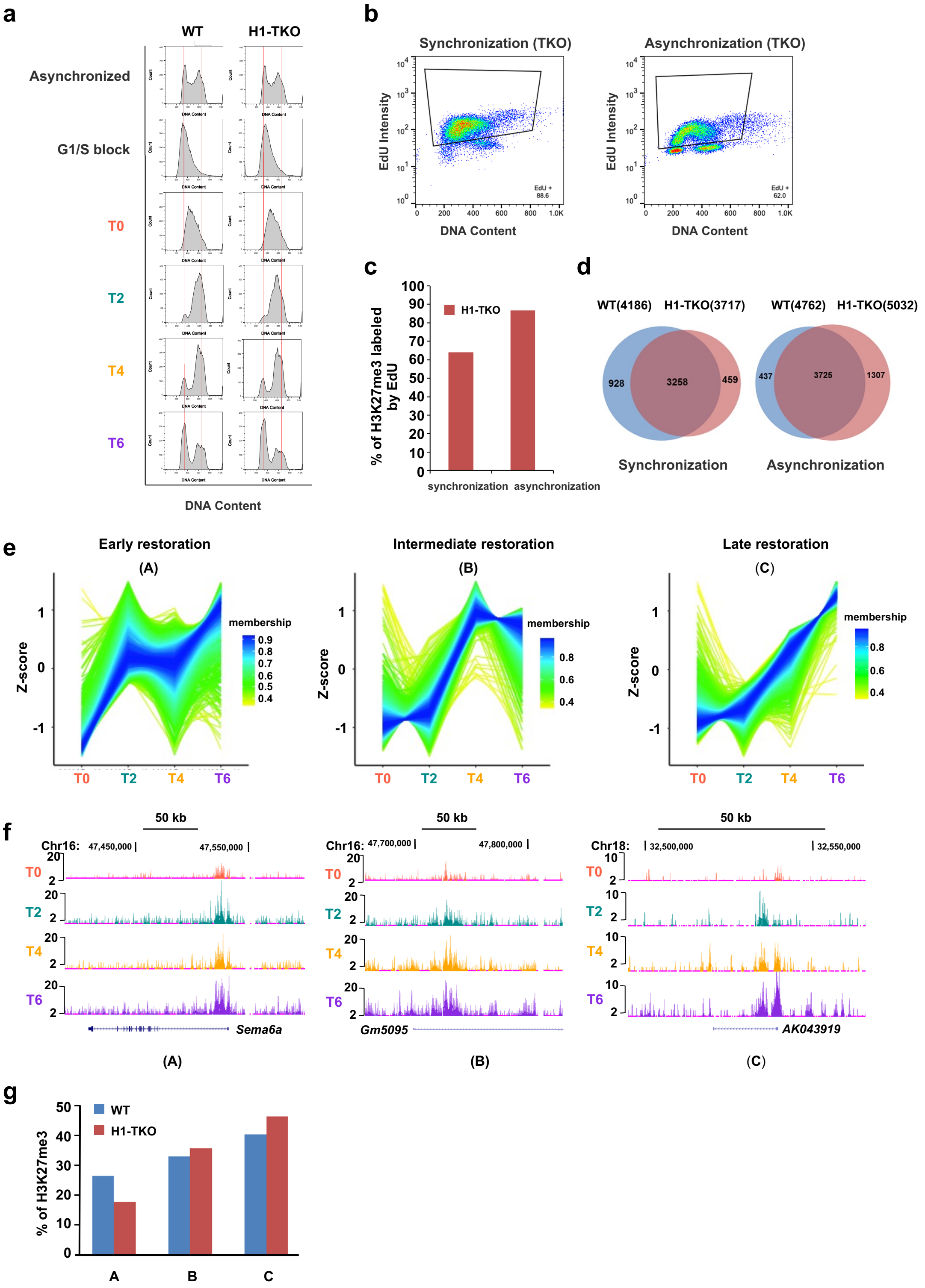

Supplementary Fig.4 H1-mediated chromatin compaction facilitates the restoration of H3K27me3 following DNA replication.

(**a**) Cell cycle progression was monitored by FACS analysis of DNA content in wide type and H1-TKO mESCs. (**b**) Representative flow cytometry profiles of EdU-labeled cells in nascent samples of synchronized and asynchronous H1-TKO mESCs. (**c**) Bar

plots showing the % of EdU-coverage of H3K27me3 peaks in synchronized and asynchronous H1-TKO mESCs compared with all H3K27me3 peaks. (**d**) Venn diagram showing the overlapping of EdU-labeled H3K27me3 peaks in WT (blue) and H1-TKO (orange) mESCs at synchronized (left) and asynchronous (right) conditions. (**e**) Clusters of the restoration pattern of H3K27me3 at its enriched peak regions in H1-TKO mESCs using time series cluster analysis. (**f**) Snap shot of tracks from the 3 clusters of H3K27me3 restoration pattern in H1-TKO mESCs. (**g**) Proportion of H3K27me3 peaks in wide type and H1-TKO mESCs among 3 clusters (A-C). Source data are provided as a Source Data file.

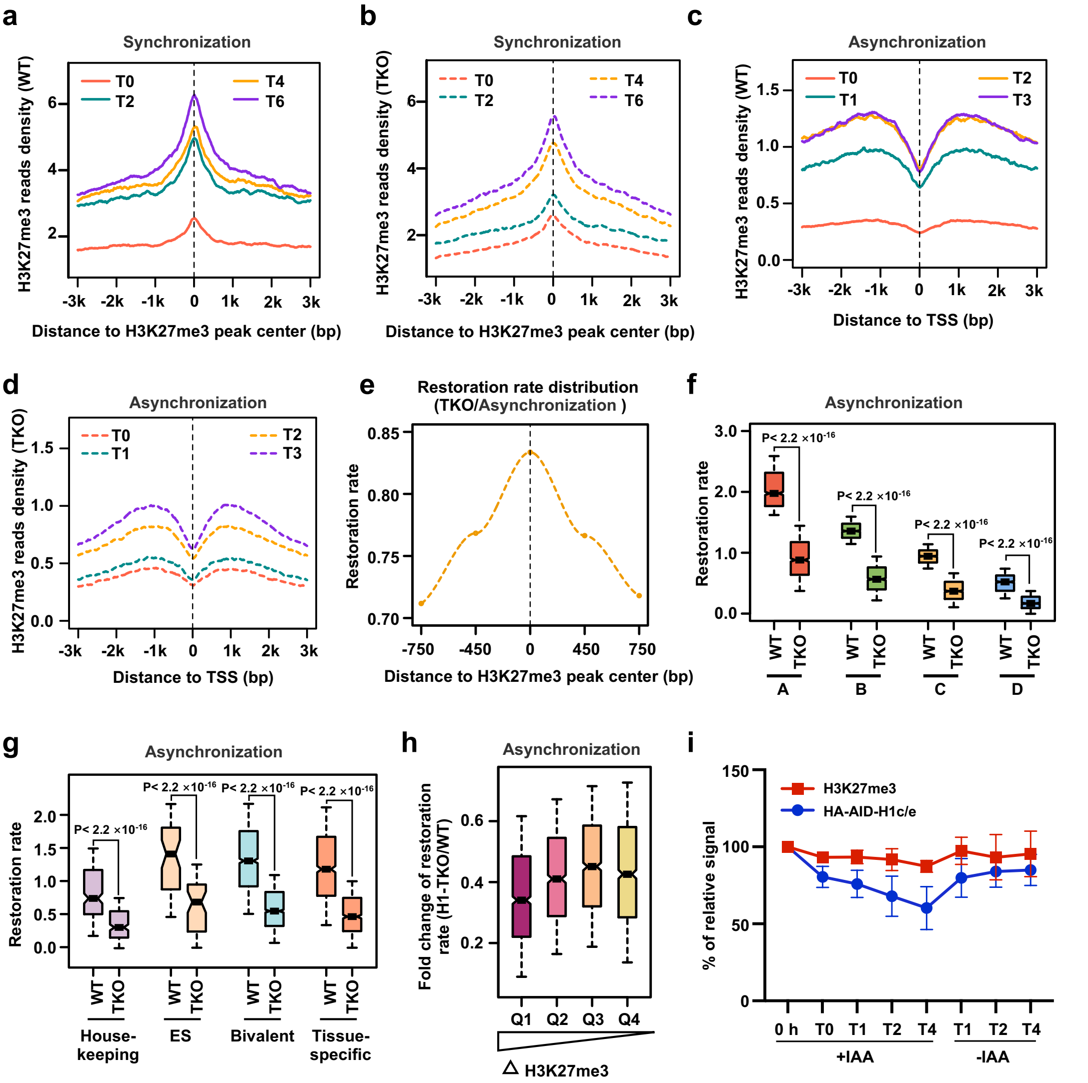

Supplementary Fig.5 H1-mediated chromatin compaction facilitates the restoration of H3K27me3 following DNA replication.

(**a, b**) Average profiles of H3K27me3 ChOR-seq signals post DNA replication at different time points in wild type (a) and H1-TKO (b) synchronized mESCs, respectively, centered at the EdU-labeled peak summit of H3K27me3. (**c, d**) Average profiles of H3K27me3 ChOR-seq signals post DNA replication at different time points in wild type (a) and H1-TKO (b) asynchronous mESCs, respectively, centered at EdU-labeled and H3K27me3-enriched gene TSSs. The ChOR-seq assay in asynchronous mESCs was performed twice. (a-d) is quantitated using reference-adjusted reads per kilobase per million (RPKM). (**e**) Average profiles of restoration rate of H3K27me3 in the asynchronous H1-TKO mESCs, plotted across ± 750 bp EdU-labeled H3K27me3

peak summit. Calculated using 1.5 kb windows with a 300 bp step, please see details in Methods section. (**f**) Boxplot showing the comparison of restoration rate of H3K27me3 among cluster A-D in the asynchronous and H1-TKO mESCs (n= 1115 for A, n= 1116 for B, n= 1115 for C, n= 1116 for D). The P values are calculated according to Wilcoxon signed-rank test (two-sided, $p<2.2\times10^{-16}$). (**g**) Boxplot showing the restoration rate of H3K27me3 at house-keeping, ES, bivalent and tissue-specific gene cluster regions that enrich H3K27me3 marks (WT versus H1-TKO asynchronous mESCs) (n= 200 for House-keeping genes, n= 49 for ES genes, n= 1488 for Bivalent genes, n= 881 for Tissue-specific genes). The P values are calculated according to Wilcoxon signed-rank test (two-sided, $p<2.2\times10^{-16}$). (**h**) Boxplot showing the change of restoration rate of H3K27me3 in the asynchronous H1-TKO mESCs compared with that in wild-type mESCs among cluster Q1-Q4 (n= 1739 for Q1, n= 1739 for Q2, n= 1739 for Q3, n= 1740 for Q4). (**i**) Quantitative signals of H1c/e and H3K27me3 immunoblot by Image J, normalized to H3 signal (n=3 biologically independent experiments). Data represents mean ± S.D. The box plots (f-h) include the median line (median value indicated), the box denotes the interquartile range (IQR), whiskers denote the rest of the data distribution, and outliers are denoted by points greater than ±1.5×IQR. Source data are provided as a Source Data file.

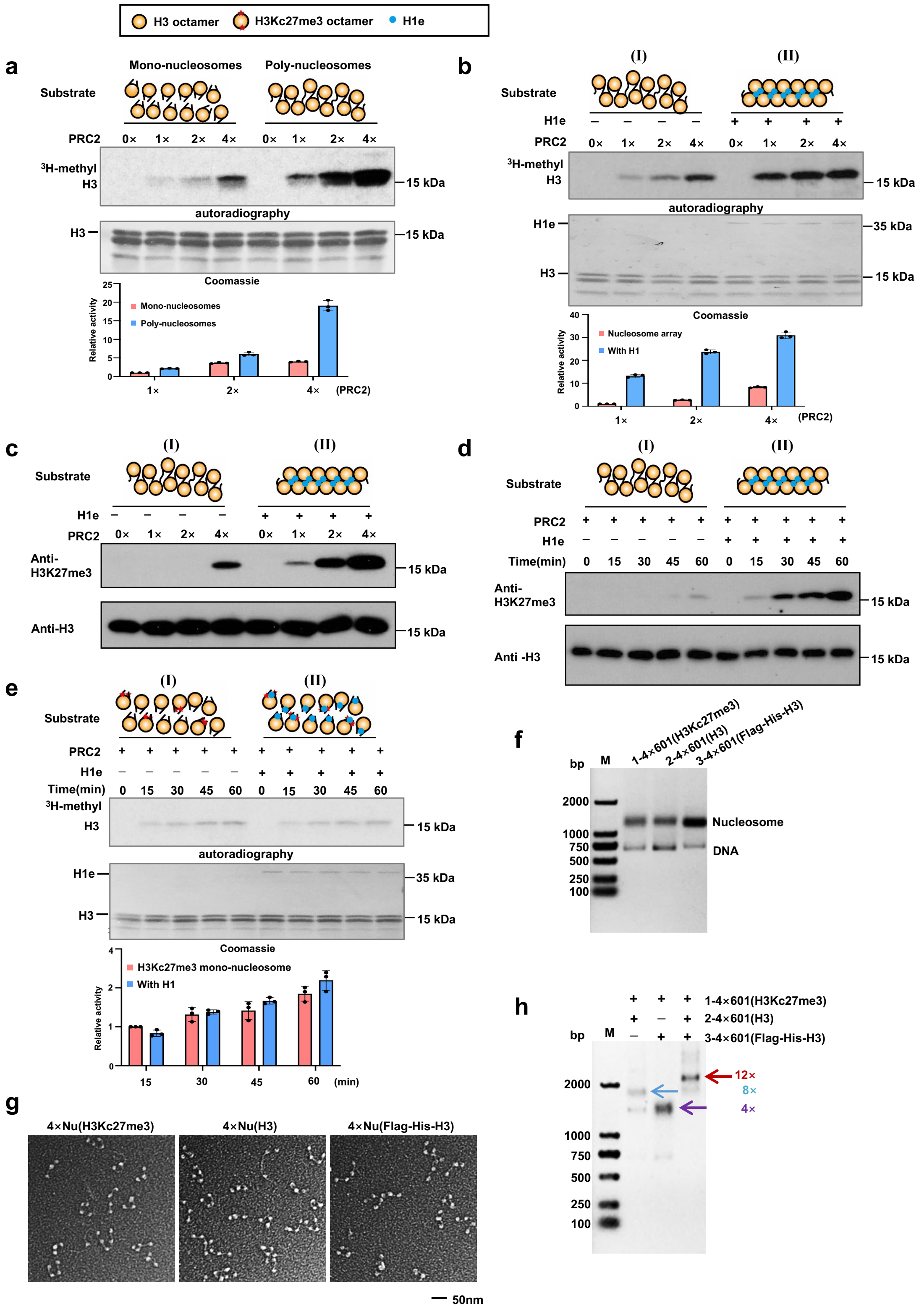

Supplementary Fig.6 H1-mediated chromatin compaction promotes the propagation of H3K27me3 via a nucleosome-nucleosome pairing mechanism.

(**a-e**) *In vitro* histone methyltransferase assays of PRC2, the signals of H3K27me3 products were detected by ^{3}H-methyl autoradiography or immunoblots, quantitative

analysis of ^{3}H signals by liquid scintillation. The results are representative of *n*=3 biologically independent experiments. The data represent means ± S.D. The amount of substrates was detected by coomassie staining. In **a**, ^{3}H-methyl autoradiography showing the activity of PRC2 on mono-nucleosome and poly-nucleosome substrates. In **b**, ^{3}H-methyl autoradiography showing the activity of PRC2 on nucleosome arrays and H1-compacted chromatin substrates. In **c**, immunoblots showing the activity of PRC2 on poly-nucleosome and H1-compacted chromatin substrates. In **d**, immunoblots showing the activity of PRC2 on poly-nucleosome and H1-compacted chromatin substrates. The reactions were incubated for indicated time at 30℃. In **e**, ^{3}H-methyl autoradiography showing the activity of PRC2 on mono-nucleosome and H1-bound mono-nucleosome substrates. (**f**) Representative 1.2% agarose gel electrophoresis of individual tetra-nucleosomes that contained H3Kc27me3, H3 and Flag-His-H3, respectively. The data are representative of *n* = 3 biologically independent experiments. (**g**) Representative EM images of the tetra-nucleosomes for ligation assays. Scale bar is 50 nm. The data are representative of *n* = 3 biologically independent experiments. (**h**) Representative 1.2% agarose gel electrophoresis of 12× poly-nucleosomes generated by sequential ligation with tetra-nucleosomes out of 3 independent experiments. Source data are provided as a Source Data file.

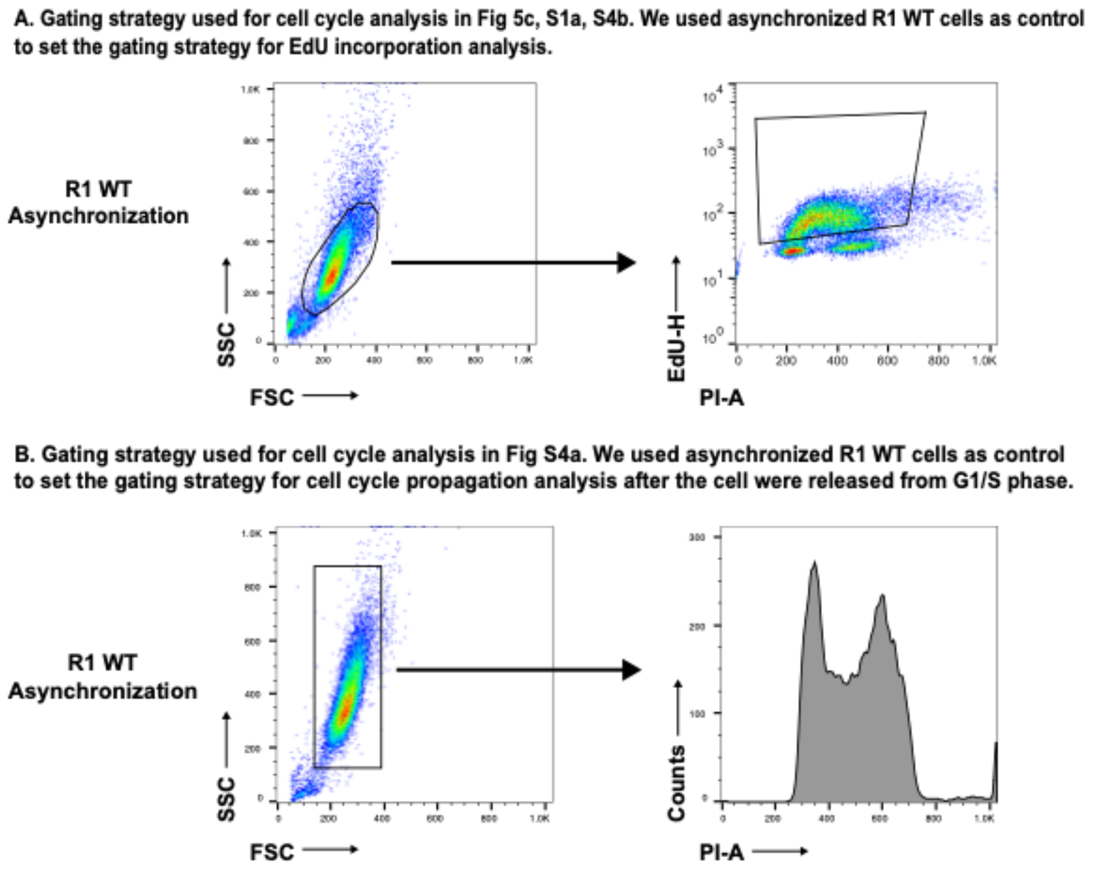

Supplementary Fig.7 Gating strategy
