# Supplementary material for: Histone H1 facilitates restoration of H3K27me3 during DNA replication by chromatin compaction
Source: Nat Commun. 2023 Jul 10;14:4081. doi: 10.1038/s41467-023-39846-y (PMC10333366; doi:10.1038/s41467-023-39846-y)
Supplement: Supplementary file 3 — Reporting Summary [file 41467_2023_39846_MOESM3_ESM.pdf]

nature portfolio

Corresponding author(s): Guohong Li

Last updated by author(s): Jun 20, 2023

Reporting Summary

Nature Portfolio wishes to improve the reproducibility of the work that we publish. This form provides structure for consistency and transparency in reporting. For further information on Nature Portfolio policies, see our Editorial Policies and the Editorial Policy Checklist.

Statistics

For all statistical analyses, confirm that the following items are present in the figure legend, table legend, main text, or Methods section.

n/a	Confirmed	
☐	☒	The exact sample size (*n*) for each experimental group/condition, given as a discrete number and unit of measurement
☐	☒	A statement on whether measurements were taken from distinct samples or whether the same sample was measured repeatedly
☐	☒	The statistical test(s) used AND whether they are one- or two-sided<br>*Only common tests should be described solely by name; describe more complex techniques in the Methods section.*
☒	☐	A description of all covariates tested
☐	☒	A description of any assumptions or corrections, such as tests of normality and adjustment for multiple comparisons
☐	☒	A full description of the statistical parameters including central tendency (e.g. means) or other basic estimates (e.g. regression coefficient) AND variation (e.g. standard deviation) or associated estimates of uncertainty (e.g. confidence intervals)
☐	☒	For null hypothesis testing, the test statistic (e.g. *F*, *t*, *r*) with confidence intervals, effect sizes, degrees of freedom and *P* value noted<br>*Give P values as exact values whenever suitable.*
☒	☐	For Bayesian analysis, information on the choice of priors and Markov chain Monte Carlo settings
☐	☒	For hierarchical and complex designs, identification of the appropriate level for tests and full reporting of outcomes
☐	☒	Estimates of effect sizes (e.g. Cohen's *d*, Pearson's *r*), indicating how they were calculated

Our web collection on statistics for biologists contains articles on many of the points above.

Software and code

Policy information about availability of computer code

Data collection

EM images were collected with FEI Tecnai G2 Spirit 120 kV transmission electron microscope.
ChIP-seq data of single-read and paired-end were collected with Illumina Hiseq2500/Nova seq and Hiseq6000/Nova seq, respectively.
ChOR-seq data of paired-end were colledted with Illumina NovaSeq 6000 seq
RNA-seq data of paired-end were collected with Illumina Hiseq6000/Nova seq.
ChIP-qPCR data were collected with StepOnePlusTM realtime PCR system/Thermofisher.
Flow cytometry experiments were performed with BD FACS Calibur. Data was collected by CellQuest. Data was analyzed by Flowjo 10.

Data analysis

EM images were processed with Photoshop.
For ChIP-seq data analysis: Reads was mapped to mm9 by bowtie2 (version 2.2.5), filtered with Samtools (version 1.2.1). Peaks were detected with MACS (version1.4.2). Peak overlapping was analyzed with Bed tools (version 2.17.0). Homer was used to annotate the peaks and count the read density.Data tracks were viewed with UCSC. Heat maps were generated using Java TreeView .Hierarchical clustering was performed using unweighted pair group method with the distance between profiles computed as pearson's correlation coefficients by ggplot2.
ChOR-seq data were mapped to the Mus musculus genome assembly (mm9) and D. melanogaster genome assembly (dm6) using bowtie2, with the default parameters. Low quality reads and PCR duplicates were removed by samtools and only uniquely mapped reads which mapping to a unique genomic location and strand were kept. Reads were normalized with fly reference as previous study. The enriched regions (peaks) were detected using MACS. Time series cluster analysis was performed by TCseq package. Hilbert curves of H3K27me3 ChOR-seq data visualized by R package HilbertCurve.
RNA-seq reads were mapped to the Mus musculus mm9 gene annotation model using Subread with the default parameters, and the only uniquely mapped reads were used for further analysis. The differential expression between samples was performed with featureCounts and R package DESeq2. Gene enrichment for one gene group was analyzed by the Metascape.

ChIP-qPCR data were analyzed with Microsoft Excel for Mac 16.59 and plotted with GraphPad Prism 8.
Flow cytometry data analysis was performed by Flowjo 10.

For manuscripts utilizing custom algorithms or software that are central to the research but not yet described in published literature, software must be made available to editors and reviewers. We strongly encourage code deposition in a community repository (e.g. GitHub). See the Nature Portfolio guidelines for submitting code & software for further information.

Data

Policy information about availability of data

All manuscripts must include a data availability statement. This statement should provide the following information, where applicable:

- Accession codes, unique identifiers, or web links for publicly available datasets
- A description of any restrictions on data availability
- For clinical datasets or third party data, please ensure that the statement adheres to our policy

ChIP-seq, ChOR-seq and RNA-seq data that generated in this study have been deposited in the GEO under accession code GSE192984[https://www.ncbi.nlm.nih.gov/geo/query/acc.cgi?acc=GSE192984]. Previously published ChIP-seq data that were re-analyzed here are available in SRA under the accession codes: SRX7585130[https://www.ncbi.nlm.nih.gov/sra/SRX7585130](MNase-seq), SRR1703159[https://www.ncbi.nlm.nih.gov/sra/?term=SRR1703159](ATAC-seq), SRR579162[https://www.ncbi.nlm.nih.gov/sra/?term=SRR579162](DNase-seq), SRR925639[https://www.ncbi.nlm.nih.gov/sra/?term=SRR925639] (H3K4me1 ChIP-seq), SRR1044607[https://www.ncbi.nlm.nih.gov/sra/?term=SRR1044607] (H3K4me2 ChIP-seq), SRR925640[https://www.ncbi.nlm.nih.gov/sra/?term=SRR925640] (H3K4me3 ChIP-seq), SRR1648485[https://www.ncbi.nlm.nih.gov/sra/?term=SRR1648485] (H3K9me1 ChIP-seq), SRR1130791[https://www.ncbi.nlm.nih.gov/sra/?term=SRR1130791] (H3K9me2 ChIP-seq), SRR925641[https://www.ncbi.nlm.nih.gov/sra/?term=SRR925641](H3K9me3 ChIP-seq), SRX8373531[https://www.ncbi.nlm.nih.gov/sra/?term=SRX8373531] (H3 ChIP-seq), SRX8373532[https://www.ncbi.nlm.nih.gov/sra/?term=SRX8373532] (H4 ChIP-seq), SRX4386198[https://www.ncbi.nlm.nih.gov/sra/?term=SRX4386198](H3.3 ChIP-seq), SRX111869 [https://www.ncbi.nlm.nih.gov/sra/?term=SRX111869](H2A.Z ChIP-seq), SRR2673297[https://www.ncbi.nlm.nih.gov/sra/?term=SRR2673297] (H3K27me3 ChIP-seq), SRR925647[https://www.ncbi.nlm.nih.gov/sra/?term=SRR925647] (H3K36me3 ChIP-seq), SRX317656[https://www.ncbi.nlm.nih.gov/sra/?term=SRX317656] (H3K27ac ChIP-seq), SRR1799183[https://www.ncbi.nlm.nih.gov/sra/?term=SRR1799183] (H3K56ac ChIP-seq), SRR1635424[https://www.ncbi.nlm.nih.gov/sra/?term=SRR1635424] (H3K79me2 ChIP-seq). Source data are provided with this paper.

Research involving human participants, their data, or biological material

Policy information about studies with human participants or human data. See also policy information about sex, gender (identity/presentation), and sexual orientation and race, ethnicity and racism.

Reporting on sex and gender	N/A
Reporting on race, ethnicity, or other socially relevant groupings	N/A
Population characteristics	N/A
Recruitment	N/A
Ethics oversight	N/A

Note that full information on the approval of the study protocol must also be provided in the manuscript.

Field-specific reporting

Please select the one below that is the best fit for your research. If you are not sure, read the appropriate sections before making your selection.

☒ Life sciences ☐ Behavioural & social sciences ☐ Ecological, evolutionary & environmental sciences

For a reference copy of the document with all sections, see nature.com/documents/nr-reporting-summary-flat.pdf

Life sciences study design

All studies must disclose on these points even when the disclosure is negative.

Sample size	No statistical method was used to predetermine sample size. Sample size for 3 biological replicates of Western Blots, EM, ChIP-qPCR and 2 biological replicates of key ChIP-seq, ChOR-seq and RNA-seq experiments in this study were used according to common practice in the field. Sample size of each gene group all>30, which is sufficient for statistical analysis.
Data exclusions	No data were excluded from the analyses.
Replication	Western Blots, EM, ChIP-qPCR were independently replicated 3 times, as stated in the figure legend, and showed similar results.<br>ChIP-seq, ChOR-seq and RNA-seq experiments were performed independently in duplicates, as stated in the figure legend, and showed similar results.

Randomization	Experimental materials were not divided into random subgroups. No randomization was involved in this study.
Blinding	Blinding was not performed due to the unambiguous nature of measurements and systematic analyses used in these experiments.

Reporting for specific materials, systems and methods

We require information from authors about some types of materials, experimental systems and methods used in many studies. Here, indicate whether each material, system or method listed is relevant to your study. If you are not sure if a list item applies to your research, read the appropriate section before selecting a response.

Materials & experimental systems

n/a	Involved in the study
☐	☒ Antibodies
☐	☒ Eukaryotic cell lines
☒	☐ Palaeontology and archaeology
☒	☐ Animals and other organisms
☒	☐ Clinical data
☒	☐ Dual use research of concern
☒	☐ Plants

Methods

n/a	Involved in the study
☐	☒ ChIP-seq
☐	☒ Flow cytometry
☒	☐ MRI-based neuroimaging

Antibodies

Antibodies used

Antibodies for western blots: Anti-Histone H1, Active motif, 61202, polyclone, 1:2000;
Anti-Suz12, Cell signaling technology, 3737S,1:2000, polyclone;
Anti-trimethyl-Histone H3 (Lys27)(C36B11),Cell signaling technology, 9733S, 1:2000;
Anti-Histone H3, Abcam, ab1791, polyclone, 1:5000;
Anti-Histone H3 (trimethyl K9), Abcam, ab8898, polyclone, 1:2000;
Anti-Histone H3 (trimethyl K4), Abcam, ab8580, polyclone, 1:2000;
Anti-Histone H3 (dimethyl K4), Millipore,07-030,polyclone, 1:2000;
Anti-Histone H3 (trimethyl K36), Abcam, ab9050, polyclone, 1:2000;
Anti-Histone H3 (Dimethyl Lys9), NOVUS, NB21-1072S, polyclone, 1:2000;
Anti-Histone H4 (di methyl K20), Abcam, ab9052, polyclone, 1:2000;
Anti-Histone H4 (tri methyl K20),Abcam, ab9053, polyclone, 1:2000;
Anti-Histone H3 (di methyl K36), Abcam,ab9049, polyclone,1:2000;
Anti-HA, Sigma, H3663, HA-7,Monoclonal , 1:2000;
Antibodies for ChIP: Anti-HA, Sigma, H3663, HA-7,Monoclonal ;
Anti-trimethyl-Histone H3 (Lys27)(C36B11),Cell signaling technology,9733S;
Anti-V5 Tag, Gene-Protein Link, P01L075.

Validation

All antibodies used in this study have been extensively validated by the manufacturers (see manufacturer web site for details).
Anti-Suz12:https://www.cellsignal.cn/products/primary-antibodies/suz12-d39f6-xp-rabbit-mab/3737?site-search-type=Products&N=4294956287&Ntt=3737s&fromPage=plp&_requestid=1269203
Anti-trimethyl-Histone H3 (Lys27): https://www.cellsignal.cn/products/primary-antibodies/tri-methyl-histone-h3-lys27-c36b11-rabbit-mab/9733?site-search-type=Products&N=4294956287&Ntt=9733s&fromPage=plp&_requestid=1269545
Anti-Histone H1: https://www.activemotif.com.cn/catalog/details/61201/histone-h1-antibody-pab-2
Anti-Histone H3, Abcam: https://www.abcam.cn/products/primary-antibodies/histone-h3-antibody-nuclear-marker-and-chip-grade-ab1791.html
Anti-Histone H3 (trimethyl K4), Abcam: https://www.abcam.cn/products/primary-antibodies/histone-h3-tri-methyl-k4-antibody-chip-grade-ab8580.html
Anti-Histone H3 (trimethyl K9), Abcam:https://www.abcam.cn/products/primary-antibodies/histone-h3-tri-methyl-k9-antibody-chip-grade-ab8898.html
Anti-Histone H3 (trimethyl K36), Abcam:https://www.abcam.cn/products/primary-antibodies/histone-h3-tri-methyl-k36-antibody-chip-grade-ab9050.html
Anti-Histone H4 (tri methyl K20),Abcam: https://www.abcam.cn/products/primary-antibodies/histone-h4-tri-methyl-k20-antibody-chip-grade-ab9053.html
Anti-Histone H4 (di methyl K20), Abcam: https://www.abcam.cn/products/primary-antibodies/histone-h4-di-methyl-k20-antibody-ab9052.html
Anti-Histone H3 (Dimethyl Lys9), NOVUS:https://www.novusbio.com/products/histone-h3-antibody_nb21-1072
Anti-Histone H3 (di methyl K36), Abcam:https://www.abcam.cn/products/primary-antibodies/histone-h3-di-methyl-k36-antibody-chip-grade-ab9049.html
Anti-HA, Sigma: https://www.sigmaaldrich.cn/CN/zh/product/sigma/h3663
Anti-V5 Tag, Gene-Protein Link:http://www.gplink.com.cn/biofriendship/a/chanpinzhongxin/chanpinchaxun/index.html?kw=V5+RABBIT&kwtype=product_name&companyid=0
Anti-Histone H3 (dimethyl K4), Millipore: https://www.sigmaaldrich.cn/CN/zh/product/mm/07-030

Eukaryotic cell lines

Policy information about cell lines and Sex and Gender in Research

Cell line source(s)	All the mouse embryonic stem cell lines generated in our lab are with a genetic background of mouse embryonic stem cell line R1, which is sourced from ATCC. The Drosophila S2 cell line is sourced from ATCC.
Authentication	Cell lines of mouse embryonic stem cell generated through gene editing were validated by sequencing and Identity of cell line was frequently checked by the morphological features. We did not edit Drosophila S2 cell line.
Mycoplasma contamination	Cells were monitored and found negative for mycoplasma.
Commonly misidentified lines (See ICLAC register)	N/A

Plants

Seed stocks	N/A
Novel plant genotypes	N/A
Authentication	N/A

ChIP-seq

Data deposition

☒ Confirm that both raw and final processed data have been deposited in a public database such as GEO.

☒ Confirm that you have deposited or provided access to graph files (e.g. BED files) for the called peaks.

Data access links
May remain private before publication.

ChIP-seq, ChOR-seq and RNA-seq data that generated in this study have been deposited in the GEO under accession code GSE192984[https://www.ncbi.nlm.nih.gov/geo/query/acc.cgi?acc=GSE192984]. Previously published ChIP-seq data that were re-analyzed here are available in SRA under the accession codes: SRX7585130[https://www.ncbi.nlm.nih.gov/sra/SRX7585130](MNase-seq), SRR1703159[https://www.ncbi.nlm.nih.gov/sra/?term=SRR1703159](ATAC-seq), SRR579162[https://www.ncbi.nlm.nih.gov/sra/?term=SRR579162](DNase-seq), SRR925639[https://www.ncbi.nlm.nih.gov/sra/?term=SRR925639] (H3K4me1 ChIP-seq), SRR1044607[https://www.ncbi.nlm.nih.gov/sra/?term=SRR1044607] (H3K4me2 ChIP-seq), SRR925640[https://www.ncbi.nlm.nih.gov/sra/?term=SRR925640] (H3K4me3 ChIP-seq), SRR1648485[https://www.ncbi.nlm.nih.gov/sra/?term=SRR1648485] (H3K9me1 ChIP-seq), SRR1130791[https://www.ncbi.nlm.nih.gov/sra/?term=SRR1130791] (H3K9me2 ChIP-seq), SRR925641[https://www.ncbi.nlm.nih.gov/sra/?term=SRR925641](H3K9me3 ChIP-seq), SRX8373531[https://www.ncbi.nlm.nih.gov/sra/?term=SRX8373531] (H3 ChIP-seq), SRX8373532[https://www.ncbi.nlm.nih.gov/sra/?term=SRX8373532] (H4 ChIP-seq), SRX4386198[https://www.ncbi.nlm.nih.gov/sra/?term=SRX4386198](H3.3 ChIP-seq), SRX111869 [https://www.ncbi.nlm.nih.gov/sra/?term=SRX111869](H2A.Z ChIP-seq), SRR2673297[https://www.ncbi.nlm.nih.gov/sra/?term=SRR2673297] (H3K27me3 ChIP-seq), SRR925647[https://www.ncbi.nlm.nih.gov/sra/?term=SRR925647] (H3K36me3 ChIP-seq), SRX317656[https://www.ncbi.nlm.nih.gov/sra/?term=SRX317656] (H3K27ac ChIP-seq), SRR1799183[https://www.ncbi.nlm.nih.gov/sra/?term=SRR1799183] (H3K56ac ChIP-seq), SRR1635424[https://www.ncbi.nlm.nih.gov/sra/?term=SRR1635424] (H3K79me2 ChIP-seq). Source data are provided with this paper.

Files in database submission

GSM5770164 ChIP-seq_WT_H3K27me3_rep1
GSM5770165 ChIP-seq_WT_H3K27me3_rep2
GSM5770166 ChIP-seq_H1KO_H3K27me3_rep1
GSM5770167 ChIP-seq_H1KO_H3K27me3_rep2
GSM5770168 ChIP-seq_WT_H1
GSM5770169 ChOR-seq_WT_T0_rep1
GSM5770170 ChOR-seq_WT_T0_rep2
GSM5770171 ChOR-seq_WT_T2_rep1
GSM5770172 ChOR-seq_WT_T2_rep2
GSM5770173 ChOR-seq_WT_T4_rep1
GSM5770174 ChOR-seq_WT_T4_rep2
GSM5770175 ChOR-seq_WT_T6_rep1
GSM5770176 ChOR-seq_WT_T6_rep2
GSM5770177 ChOR-seq_H1KO_T0_rep1
GSM5770178 ChOR-seq_H1KO_T0_rep2
GSM5770179 ChOR-seq_H1KO_T2_rep1
GSM5770180 ChOR-seq_H1KO_T2_rep2
GSM5770181 ChOR-seq_H1KO_T4_rep1
GSM5770182 ChOR-seq_H1KO_T4_rep2
GSM5770183 ChOR-seq_H1KO_T6_rep1
GSM5770184 ChOR-seq_H1KO_T6_rep2
GSM5770185 RNA-seq_WT_rep1
GSM5770186 RNA-seq_WT_rep2

GSM5770187 RNA-seq_H1KO_rep1
GSM5770188 RNA-seq_H1KO_rep2
GSM6722604 ChIP-seq_WT_HA_tetR_EED_targeting_H3K27me3
GSM6722605 ChIP-seq_WT_HA_tetR_EZH2_targeting_H3K27me3
GSM6722606 ChIP-seq_H1KO_HA_tetR_EED_targeting_H3K27me3
GSM6722607 ChIP-seq_H1KO_HA_tetR_EZH2_targeting_H3K27me3
GSM6722608 ChOR-seq_WT_T0_asynchrony
GSM6722609 ChOR-seq_WT_T1_asynchrony
GSM6722610 ChOR-seq_WT_T2_asynchrony
GSM6722611 ChOR-seq_WT_T3_asynchrony
GSM6722612 ChOR-seq_H1KO_T0_asynchrony
GSM6722613 ChOR-seq_H1KO_T1_asynchrony
GSM6722614 ChOR-seq_H1KO_T2_asynchrony
GSM6722615 ChOR-seq_H1KO_T3_asynchrony
GSM6722616 ChOR-seq_AID_WT_T0_asynchrony
GSM6722617 ChOR-seq_AID_increase_WT_T1_asynchrony
GSM6722618 ChOR-seq_AID_increase_WT_T2_asynchrony
GSM6722619 ChOR-seq_AID_increase_WT_T4_asynchrony
GSM6722620 ChOR-seq_AID_decrease_WT_T1_asynchrony
GSM6722621 ChOR-seq_AID_decrease_WT_T2_asynchrony
GSM6722622 ChOR-seq_AID_decrease_WT_T4_asynchrony
GSM6722623 Streptavidin_pull-down_WT_synchronization
GSM6722624 Streptavidin_pull-down_H1KO_synchronization
GSM6722625 Streptavidin_pull-down_WT_asynchrony
GSM6722626 Streptavidin_pull-down_H1KO_asynchrony
GSM7123855 ChIP-seq_WT_HA_tetR_EED_targeting_H3K27me3_rep2
GSM7123856 ChIP-seq_WT_HA_tetR_EZH2_targeting_H3K27me3_rep2
GSM7123857 ChIP-seq_H1KO_HA_tetR_EED_targeting_H3K27me3_rep2
GSM7123858 ChIP-seq_H1KO_HA_tetR_EZH2_targeting_H3K27me3_rep2
GSM7123859 ChOR-seq_WT_T0_asynchrony_rep2
GSM7123860 ChOR-seq_WT_T1_asynchrony_rep2
GSM7123861 ChOR-seq_WT_T2_asynchrony_rep2
GSM7123862 ChOR-seq_WT_T3_asynchrony_rep2
GSM7123863 ChOR-seq_H1KO_T0_asynchrony_rep2
GSM7123864 ChOR-seq_H1KO_T1_asynchrony_rep2
GSM7123865 ChOR-seq_H1KO_T2_asynchrony_rep2
GSM7123866 ChOR-seq_H1KO_T3_asynchrony_rep2
GSM7123867 ChOR-seq_AID_WT_T0_asynchrony_rep2
GSM7123868 ChOR-seq_AID_increase_WT_T1_asynchrony_rep2
GSM7123869 ChOR-seq_AID_increase_WT_T2_asynchrony_rep2
GSM7123870 ChOR-seq_AID_increase_WT_T4_asynchrony_rep2
GSM7123871 ChOR-seq_AID_decrease_WT_T1_asynchrony_rep2
GSM7123872 ChOR-seq_AID_decrease_WT_T2_asynchrony_rep2
GSM7123873 ChOR-seq_AID_decrease_WT_T4_asynchrony_rep2
GSM7123874 Streptavidin_pull-down_WT_synchronization_rep2
GSM7123875 Streptavidin_pull-down_H1KO_synchronization_rep2
GSM7123876 Streptavidin_pull-down_WT_asynchrony_rep2
GSM7123877 Streptavidin_pull-down_H1KO_asynchrony_rep2

Genome browser session
(e.g. UCSC)

no longer applicable

Methodology

Replicates

ChIP-seq, ChOR-seq and RNA-seq experiments were performed in duplicates.
H1 ChIP-seq was performed once.

Sequencing depth

ChIP-seq_WT_H3K27me3_rep1, single-end, read number: 43452257, read length: 50 bp
ChIP-seq_WT_H3K27me3_rep2, pair-end, read number: 33238048, read length: 150 bp
ChIP-seq_H1KO_H3K27me3_rep1, single-end, read number: 42407065, read length: 50 bp
ChIP-seq_H1KO_H3K27me3_rep2, pair-end, read number: 35268053, read length: 150 bp
ChIP-seq_WT_H1, pair-end, read number: 21270473, read length: 150 bp
ChOR-seq_WT_T0_rep1, pair-end, read number: 29181688, read length: 150 bp
ChOR-seq_WT_T0_rep2, pair-end, read number: 40519117, read length: 150 bp
ChOR-seq_WT_T2_rep1, pair-end, read number: 31282389, read length: 150 bp
ChOR-seq_WT_T2_rep2, pair-end, read number: 47465519, read length: 150 bp
ChOR-seq_WT_T4_rep1, pair-end, read number: 29086755, read length: 150 bp
ChOR-seq_WT_T4_rep2, pair-end, read number: 27100525, read length: 150 bp
ChOR-seq_WT_T6_rep1, pair-end, read number: 29646359, read length: 150 bp
ChOR-seq_WT_T6_rep2, pair-end, read number: 44903931, read length: 150 bp
ChOR-seq_H1KO_T0_rep1, pair-end, read number: 38445669, read length: 150 bp
ChOR-seq_H1KO_T0_rep2, pair-end, read number: 36431086, read length: 150 bp
ChOR-seq_H1KO_T2_rep1, pair-end, read number: 29720305, read length: 150 bp
ChOR-seq_H1KO_T2_rep2, pair-end, read number: 45934069, read length: 150 bp
ChOR-seq_H1KO_T4_rep1, pair-end, read number: 29960571, read length: 150 bp

ChOR-seq_H1KO_T4_rep2, pair-end, read number: 37983856, read length: 150 bp
ChOR-seq_H1KO_T6_rep1, pair-end, read number: 31717140, read length: 150 bp
ChOR-seq_H1KO_T6_rep2, pair-end, read number: 38308345, read length: 150 bp
RNA-seq_WT_rep1, pair-end, read number: 25521757, read length: 150 bp
RNA-seq_WT_rep2, pair-end, read number: 20717888, read length: 150 bp
RNA-seq_H1KO_rep1, pair-end, read number: 21565775, read length: 150 bp
RNA-seq_H1KO_rep2, pair-end, read number: 22792820, read length: 150 bp
ChIP-seq_WT_HA_tetR_EED_targeting_H3K27me3, pair-end, read number: 22993920, read length: 150 bp
ChIP-seq_WT_HA_tetR_EZH2_targeting_H3K27me3, pair-end, read number: 35102131, read length: 150 bp
ChIP-seq_H1KO_HA_tetR_EED_targeting_H3K27me3,pair-end, read number: 45115720, read length: 150 bp
ChIP-seq_H1KO_HA_tetR_EZH2_targeting_H3K27me3,pair-end, read number: 34574724, read length: 150 bp
ChOR-seq_WT_T0_asynchrony,pair-end, read number: 37249500, read length: 150 bp
ChOR-seq_WT_T1_asynchrony,pair-end, read number: 41460372, read length: 150 bp
ChOR-seq_WT_T2_asynchrony,pair-end, read number: 46316860, read length: 150 bp
ChOR-seq_WT_T3_asynchrony,pair-end, read number: 45240907, read length: 150 bp
ChOR-seq_H1KO_T0_asynchrony,pair-end, read number: 48135032, read length: 150 bp
ChOR-seq_H1KO_T1_asynchrony,pair-end, read number: 44284972, read length: 150 bp
ChOR-seq_H1KO_T2_asynchrony,pair-end, read number: 60855475, read length: 150 bp
ChOR-seq_H1KO_T3_asynchrony,pair-end, read number: 48136626, read length: 150 bp
ChOR-seq_AID_WT_T0_asynchrony,pair-end, read number: 37173810, read length: 150 bp
ChOR-seq_AID_increase_WT_T1_asynchrony,pair-end, read number: 30170362, read length: 150 bp
ChOR-seq_AID_increase_WT_T2_asynchrony,pair-end, read number: 34222781, read length: 150 bp
ChOR-seq_AID_increase_WT_T4_asynchrony,pair-end, read number: 33176197, read length: 150 bp
ChOR-seq_AID_decrease_WT_T1_asynchrony,pair-end, read number: 31970681, read length: 150 bp
ChOR-seq_AID_decrease_WT_T2_asynchrony,pair-end, read number: 47969893, read length: 150 bp
ChOR-seq_AID_decrease_WT_T4_asynchrony,pair-end, read number: 40378925, read length: 150 bp
Streptavidin_pull-down_WT_synchronization,pair-end, read number: 41686950, read length: 150 bp
Streptavidin_pull-down_H1KO_synchronization,pair-end, read number: 33573740, read length: 150 bp
Streptavidin_pull-down_WT_asynchrony,pair-end, read number: 20739135, read length: 150 bp
Streptavidin_pull-down_H1KO_asynchrony, pair-end, read number: 23970395, read length: 150 bp
ChIP-seq_WT_HA_tetR_EED_targeting_H3K27me3_rep2, pair-end, read number: 28269558, read length: 150 bp
ChIP-seq_WT_HA_tetR_EZH2_targeting_H3K27me3_rep2, pair-end, read number: 37084530, read length: 150 bp
ChIP-seq_H1KO_HA_tetR_EED_targeting_H3K27me3_rep2,pair-end, read number: 17029428, read length: 150 bp
ChIP-seq_H1KO_HA_tetR_EZH2_targeting_H3K27me3_rep2,pair-end, read number: 23309783, read length: 150 bp
ChOR-seq_WT_T0_asynchrony_rep2,pair-end, read number: 42220705, read length: 150 bp
ChOR-seq_WT_T1_asynchrony_rep2,pair-end, read number: 34332770, read length: 150 bp
ChOR-seq_WT_T2_asynchrony_rep2,pair-end, read number: 33957677, read length: 150 bp
ChOR-seq_WT_T3_asynchrony_rep2,pair-end, read number: 33193809, read length: 150 bp
ChOR-seq_H1KO_T0_asynchrony_rep2,pair-end, read number: 37764242, read length: 150 bp
ChOR-seq_H1KO_T1_asynchrony_rep2,pair-end, read number: 35125487, read length: 150 bp
ChOR-seq_H1KO_T2_asynchrony_rep2,pair-end, read number: 37110312, read length: 150 bp
ChOR-seq_H1KO_T3_asynchrony_rep2,pair-end, read number: 35104802, read length: 150 bp
ChOR-seq_AID_WT_T0_asynchrony_rep2,pair-end, read number: 33834268, read length: 150 bp
ChOR-seq_AID_increase_WT_T1_asynchrony_rep2,pair-end, read number: 37756211, read length: 150 bp
ChOR-seq_AID_increase_WT_T2_asynchrony_rep2,pair-end, read number: 43044004, read length: 150 bp
ChOR-seq_AID_increase_WT_T4_asynchrony_rep2,pair-end, read number: 41893491, read length: 150 bp
ChOR-seq_AID_decrease_WT_T1_asynchrony_rep2,pair-end, read number: 43824294, read length: 150 bp
ChOR-seq_AID_decrease_WT_T2_asynchrony_rep2,pair-end, read number: 47493182, read length: 150 bp
ChOR-seq_AID_decrease_WT_T4_asynchrony_rep2,pair-end, read number: 42076917, read length: 150 bp
Streptavidin_pull-down_WT_synchronization_rep2,pair-end, read number: 33624963, read length: 150 bp
Streptavidin_pull-down_H1KO_synchronization_rep2,pair-end, read number: 32214598, read length: 150 bp
Streptavidin_pull-down_WT_asynchrony_rep2,pair-end, read number: 30107371, read length: 150 bp
Streptavidin_pull-down_H1KO_asynchrony_rep2, pair-end, read number: 42382620, read length: 150 bp

Antibodies

Anti-trimethyl-Histone H3 (Lys27)(C36B11),Cell signaling technology,9733S;
Anti-V5 Tag, Gene-Protein Link, P01L075, plyclone

Peak calling parameters

Reads were uniquely mapped to genome using bowtie2. Peaks were called using MACS (--shiftsize=75) and PeakSeq (fdr below 0.5%)

Data quality

Read with high quality were retained using Fastx_toolkit, unique reads were used for peak calling with FDR below 0.5%

Software

MACS (version 1.4.2),Sam tools (version 1.2.1),bowtie2 (version 2.2.5),Bed tools (version 2.17.0),IGV (version 2.4),Homer, Subread, featureCounts, DAVID, Java TreeView, R (version 3.0.1)

Flow Cytometry

Plots

Confirm that:

☒ The axis labels state the marker and fluorochrome used (e.g. CD4-FITC).

☒ The axis scales are clearly visible. Include numbers along axes only for bottom left plot of group (a 'group' is an analysis of identical markers).

☒ All plots are contour plots with outliers or pseudocolor plots.

☒ A numerical value for number of cells or percentage (with statistics) is provided.

Methodology

Sample preparation	We used flow cytometry only for Cell cycle analysis and EdU incorporation analysis.<br>For cell cycle analysis: R1-WT and H1-TKO mESCs were were synchronized using single thymidine block and release, respectively. Cells were harvested at indicated time points following release. Cells were fixed with 70% ethanol, and then stained with propidium iodide to asses the DNA content of the cells.<br>For EdU incorporation analysis: R1-WT, H1-TKO and H1-AID mESCs were unsynchronized or synchronized using single thymidine block. Cells were harvested immediately after EdU pulsing. Cells were fixed with 70% ethanol, and then perform click reation to add Alexa Fluor 647 to EdU. After PBST washes, cells were subsequently stained with propidium iodide to asses the DNA content of the cells.<br>Further details of the experimental procedures are provided in the Materials and Methods.
Instrument	BD FACs Calibur.
Software	ata was collected by CellQuest.<br>Data was analyzed by Flowjo 10.
Cell population abundance	At least 30000 cells were acquired for each samples.
Gating strategy	SC/SSC gate was used for gating the population of cells to exclude cell doublets and debris.<br>Asynchronous growing cell population was first run to set up the axis. All test samples were then run the identical conditions.

☒ Tick this box to confirm that a figure exemplifying the gating strategy is provided in the Supplementary Information.
